# Social Accountability Reporting for Research (SAR4Research): checklist to strengthen reporting on studies on social accountability in the literature

**DOI:** 10.1186/s12939-022-01716-2

**Published:** 2022-08-30

**Authors:** Joan Marie Kraft, Ligia Paina, Victoria Boydell, Shatha Elnakib, Andreas Sihotang, Angela Bailey, Courtney Tolmie

**Affiliations:** 1 Office of Population and Reproductive Health, United States Agency for International Development, 500 D St SW, UA-5th Floor, Washington DC, 20547 USA; 2 Department of International Health, Health Systems Program, Johns Hopkins University School of Public Health, 615 N Wolfe Street, Baltimore, MD 21205 USA; 3 School of Health and Social Care, University of Essex, Wivenhoe Park, Colchester, CO4 3SQ UK; 4 Harry S Truman School of Public Affairs, University of Missouri, 101 Middlebush Hall, Columbia, Missouri 65211 USA; 5 Accountability Research Center, American University, 4400 Massachusetts Ave NW, Washington DC, 20016 USA; 6 Wonderlight Consulting, 8342 Charlise Rd, Richmond, VA 23235 USA

**Keywords:** Reporting checklist, social accountability, evaluation, Indonesia, Uganda

## Abstract

**Background:**

An increasing number of evaluations of social accountability (SA) interventions have been published in the past decade, however, reporting gaps make it difficult to summarize findings. We developed the *Social Accountability Reporting for Research (SAR4Research)* checklist to support researchers to improve the documentation of SA processes, context, study designs, and outcomes in the peer reviewed literature and to enhance application of findings.

**Methods:**

We used a multi-step process, starting with an umbrella review of reviews on SA to identify reporting gaps. Next, we reviewed existing guidelines for reporting on behavioral interventions to determine whether one could be used in its current or adapted form. We received feedback from practitioners and researchers and tested the checklist through three worked examples using outcome papers from three SA projects.

**Results:**

Our umbrella review of SA studies identified reporting gaps in all areas, including gaps in reporting on the context, intervention components, and study methods. Because no existing guidelines called for details on context and the complex processes in SA interventions, we used CONSORT-SPI as the basis for the SAR4Research checklist, and adapted it using other existing checklists to fill gaps. Feedback from practitioners, researchers and the worked examples suggested the need to eliminate redundancies, add explanations for items, and clarify reporting for quantitative and qualitative study components.

**Conclusions:**

Results of SA evaluations in the peer-reviewed literature will be more useful, facilitating learning and application of findings, when study designs, interventions and their context are described fully in one or a set of papers. This checklist will help authors report better in peer-reviewed journal articles. With sufficient information, readers will better understand whether the results can inform accountability strategies in their own contexts. As a field, we will be better able to identify emerging findings and gaps in our understanding of SA.

## Background

Social accountability (SA) interventions, or the mechanisms and processes by which citizens and civil society groups hold the health system and its actors accountable for their commitments, are being used more frequently in health programming in developing countries. Such interventions seek to raise awareness among community members of their rights around health and gaps in services, and empower communities to engage with actors (e.g., providers) in the health system to improve health programming and health outcomes [1, 2]. SA interventions are complex, using diverse approaches and engaging diverse stakeholders in a process to understand problems (e.g., gaps in services) and identify and take actions to solve problems. Their design, implementation, and impact are also context specific, grounded in social, economic, and political realities of where they are implemented. This complexity, along with the extended pathways and time horizons for realizing community empowerment and health outcomes create evaluation challenges. Randomized controlled trials and experimental designs are not always feasible and some outcomes are not directly measurable. Evaluations, thus, use a range of study designs, including mixed methods approaches and participatory research tools to explore both health and governance-related outcomes. There is, however, little consensus on how to best evaluate SA interventions and how to estimate and measure change in outcomes.

In 2017, the World Health Organization organized a Community of Practice on Measuring Social Accountability and Health Outcomes (COP) to build consensus on outcome measures and evaluation designs; participants, including practitioners and researchers, meet annually to share experiences, methodologies, and outcomes from their work on research and evaluation; and discuss how to action research. One of the first products of the COP was a synthesis of evaluation designs for SA interventions in health to summarize common designs, research questions, and how well the designs are implemented. Based on that synthesis and discussion during the COP meeting in 2018, participants identified limited detail and inconsistent reporting across SA studies as a key gap that hinders researchers in the field from summarizing and understanding the strength of the collective evidence on SA and identify best practices for replication in other contexts, as well as key contextual factors and mechanisms relevant to implementation [3]. As a first step toward improving the level of detail and consistency in reporting across studies, the COP charged a Reporting and Guidance Working Group (including authors of this paper) to develop a reporting checklist to be used by researchers and evaluators to improve the documentation of intervention processes, context, study designs, and outcomes in the peer reviewed literature in order to facilitate cross-study comparisons, shared learning around effective SA interventions and how they can be adapted and scaled. This paper outlines the steps we took to develop the Social Accountability Reporting for Research (SAR4Research) checklist for health programming.

## Methods

We used a multi-step process to develop and refine the SAR4Research checklist, see Table 1 for the timeline for developing the checklist. Below we describe how we identified gaps in reporting, adapted existing reporting guidelines to develop the checklist, and carried out worked examples to test and revise the proposed checklist.

**Table 1:** Timeline of checklist development

**September 2017**	First meeting of COP on measuring social accountability and health outcomes and a synthesis of evaluation designs for SA interventions identified limited detail and inconsistent reporting across SA studies
**November 2018**	Reporting and Guidance Working Group (including authors of this paper) established to develop a reporting checklist for researchers and evaluators to improve documentation
**January 2019**	Reporting and Guidance Working Group undertook the umbrella review to identify the reporting gaps and reviewed reporting guidelines to inform the draft checklist
**November 2019**	Reporting and Guidance Working Group presented the draft checklist at the annual COP meeting
**May 2020**	Reporting and Guidance Working Group shared revised checklist with COP for input and to identify worked examples for testing
**August 2020**	Reporting and Guidance Working Group tested the checklist with worked examples
**November 2020**	The finalized SAR4Research checklist was disseminated at the annual COP meeting

### Developing the checklist

To develop the checklist, three authors (VB, LP, JK) carried out an umbrella review of eighteen systematic and narrative reviews of the SA literature to extract reporting limitations [4]. Our umbrella review sought to identify gaps in reporting on SA interventions in the peer-reviewed literature, and to that end we included systematic, landscaping, critical, narrative or other reviews that: included descriptions and/or results from SA interventions implemented in low- and middle-income countries, and were published or disseminated between 2010-2020. Reviews could have included SA interventions from a range of countries, covering a range of health topics and populations (e.g., rural, urban). To identify the reviews, we applied search terms related to SA (e.g., social accountability, scorecards, participatory interventions) and evaluations (e.g., program evaluation, follow-up studies, outcome evaluation) to peer-reviewed (Pubmed) and grey literature (GoogleScholar) search engines. We also requested reviews from participants in the 2018 COP meeting and received two reviews, one captured in our literature search and one that was in progress (i.e., published after the meeting) [2, 3]. Two authors (VB, JK) reviewed the abstracts, applied selection criteria and summarized the reviews, with a focus on reporting gaps. Next, we reviewed reporting guidelines, including recommendations for reporting on clinical and behavioral interventions evaluated with randomized controlled trials, quasi-experimental designs, or realist evaluations, on qualitative research, and on economic evaluations of health interventions [5–9]. We noted items included (e.g., research design) and information required for each item. We compared the reporting gaps in SA against the reporting guidelines to assess whether any existing guidelines could be adopted “as-is” for our purposes. Because none met our needs, we adapted one guideline that had been through the guideline development process for our purposes [5, 10].

We presented the first draft of the checklist at the COP meeting in 2019. Based on feedback, we revised the checklist and drafted a narrative to describe key issues for items in the checklist (e.g., explanation of mechanisms of effect). We shared the checklist and narrative, via e-mail, with CoP members in May-June 2020, and incorporated their feedback into the checklist that we tested using worked examples.

### Testing the checklist

To test the checklist we carried out three worked examples. We requested examples from COP members and purposively selected examples that: (1) evaluated SA interventions using randomized, quasi-experimental or realist evaluation, with the intent of including a mix of study designs; (2) were carried out in the last 5-7 years; (3) collected data from community members and stakeholders; and (4) reported on at least one health outcome, preferably published in a peer reviewed journal. Based on COP member recommendations, we identified one example in Uganda and two in Indonesia. For each, we engaged with principal investigators to describe the checklist development, secure their agreement to participate in testing the checklist and join us as co-authors (authors CT, AB, and AS).

The purpose of the worked examples was to assess whether items in the checklist were included in reports, and better understand study investigators’ decisions about what information they included in one or a set of papers reporting on a study. Specifically, we considered whether: (1) information called for in the checklist was included in published or grey literature manuscripts; (2) whether the checklist omitted any domains or content area that projects reported; and (3) if the information called for in the checklist was not included in published or grey literature manuscripts, whether it was included in documentation that was not published.

For each worked example, we held initial conversations with at least one study investigator to describe our process, identify published and non-published manuscripts and reports and set the stage for further discussions about the checklist (e.g., what was the checklist, the worked examples, need to revise and streamline). Then, one author (SE) conducted the data extraction and analysis, reading published and grey literature reports to identify whether items in the checklist were present and the degree to which they were covered. When checklist items were not present in papers, we discussed the reviewed internal documentation (e.g., process documentation, draft reports not yet publicly available) with the study investigators (who joined us in authoring this paper). In our discussions, these authors were able to shed light on whether the gaps could be filled (e.g., data collected, but not reported) and how they made decisions about whether they reported specific information or not. Finally, we assessed how the checklist performed within and across the worked examples (i.e., was information for each checklist element included in at least one paper/report or in project files) to revise the checklist on last time, reducing overlap and making suggestions for depth of reporting.

## Results

### Gaps in reporting on social accountability found in the umbrella review

The literature review identified reporting gaps pertaining to: conceptual underpinnings; site description; study information; intervention; context; study design; outcomes; and analyses (see Table 2) [1–3, 11–15, 18–21]. For example, few studies described how interventions were expected to work or the pathways through which the intervention would produce outcomes. Site descriptions rarely provided characteristics of organizations involved, existing social capital, and relationships between communities and leaders [1, 2, 13–15, 18, 20, 21]. In addition, few studies reported on the genesis of the intervention (e.g., grassroots, externally funded), details of the actors involved, the scale and process of implementation, the recourse mechanisms, or linkages with other efforts [2, 11–15, 19, 21]. Study designs, analyses and outcomes were not always described in sufficient detail. One explanation for this may be the complexity of SA interventions and evaluations, for which guidelines for reporting are needed. In addition, information on how funding and the relationship between implementation and evaluation teams may have influenced the evaluation were sometimes missing [12, 14, 15]. Reasons for the gaps were not always addressed in the reviews. Please see Marston et al (2020) for details of what was reported [3].

**Table 2:** Reporting gaps identified in evidence reviews

**CONCEPTUAL UNDERPINNINGS** [1, 3, 11–17]	
Lack description of:	
• Theory of change, logic model, program theory or conceptual framework with intermediate and long-range outcomes	
• Measurement of outcomes	
**SITE DESCRIPTION** [13–15, 18]	
Lack description of:	
• Minimum conditions for implementation (e.g., expertise of local organizations, nature of social capital, relationships between citizens and state)	
• Site or location (e.g., conflict or stable governance)	
**STUDY INFORMATION** [3, 11, 16, 17, 19–21]	
Lack description or statement regarding	
• Participants in research design (whether/how community was involved) and relationship between evaluation and intervention teams	
• Study design, data collection methods and protection of human subjects	
• Perspective of study (e.g., single or multi-actor)	
• Limitations of study	
• Availability of data and funding information	
• Key words relating to SA	
**INTERVENTION** [2, 11, 12, 14, 15, 18–21]	
Lack description of or statement regarding:	
• Genesis of intervention (e.g., funded short term, grassroots, systems-oriented)	
• Actors involved, at all levels (e.g., health facility, type of provider, non-state health actor, community individuals or groups), including whether and how disadvantaged groups are involved and barriers to participation (for all actors)	
• Intervention details (e.g., process, scale, interaction with context, gendered dimensions)	
• Recourse processes and effects	
• Linkages to other accountability processes or movements	
• Any social harms or unintended negative effects	
**CONTEXT** [1, 2, 11–16, 22]	
Lack description of or statement regarding:	
• Contribution of contextual conditions that influenced design and that influence outcomes, including factors that might prevent change	
• Local power relations	
• Confounding factors	
**OUTCOMES** [1, 3, 11, 13–15, 18, 19]	
Lack of description of or statement regarding:	
• Duty bearer responsiveness	
• Community outcomes	
• Longer term outcomes (e.g., sustainability)	
**ANALYSES** [11, 14, 15]	
Lack of description of or statement regarding:	
• Distinguish between outcomes of process and outcomes of evaluation.	
• Whether outcomes vary by sub-group	
• Author reflexivity	
• Respondent validation	

### Existing guidelines and the initial “Social Accountability Reporting for Research (SAR4Research)” checklist

None of the reporting guidelines we reviewed addressed all the reporting gaps that were flagged in our literature review [5–7, 9, 10, 23–30]. For example, although most called for a description of implementing partners and intervention sites, none reflected details about the power or other relationships between implementers and participants or considered the range of contextual factors that influence implementation and outcomes of SA interventions. Further, only the RAMESES guidelines for realist evaluations capture study designs that included both quantitative and qualitative designs, a characteristic of many SA evaluations [9]. Because it had recently gone through a rigorous development process and because CONSORT guidelines are routinely used in public health, we selected the CONSORT-SPI guidelines as the basis for our checklist [5, 10].

We augmented the CONSORT-SPI guidelines to capture the unique components of SA interventions and evaluations, such as accounting for diverse contextual conditions and actors, issues around equity and representation, complex, non-linear SA processes, and pathways from intermediate- to longer-term community empowerment and health outcomes. To augment the CONSORT-SPI, we drew from other relevant guidelines such as RAMESES and CICI [29, 31]. For example, we drew upon the CICI recommendations for items related to reporting on context [31]. We also added content to draw out more information related to key reporting gaps such as context, mechanisms of effect, and longer-term outcomes.

The first draft of the SAR4Research checklist contained six sections, corresponding to the typical sections of peer-reviewed articles: Title and abstract (1a-b); Introduction (2 a-d); Methods (3a; 4a-b; 5a-d; 6a-c; 7a-b); Results (8; 9; 10a-b; 11; 12a-b; 13; 14a-b; 15); Discussion (16-18); and Important information. The checklist was targeted at researchers reporting the implementation and/or evaluation of SA interventions. The checklist was designed to be applicable to various methodologies used to study SA – notably qualitative, quantitative and mixed methods approaches, as well as a range of study designs (e.g., randomized controlled trials, quasi-experimental designs, qualitative case studies). The original draft of the checklist is available by request.

### SAR4Research checklist review and testing

Feedback on the first draft of the checklist (November 2018) from COP members emphasized the need to clarify the purpose of the checklist, to streamline and reduce the number of items and redundancy across sections, and to test the checklist on available case studies to determine if all items are practical (i.e., if study teams have data to report). In addition, because the checklist is intended to be responsive to different study designs and methodologies, COP members encouraged us to enhance the description of each items to ensure that users could easily identify the items relevant to their study. We clarified the items, but did not reduce the number of items.

The revised draft of the checklist was then applied to three worked examples, including the Transparency 4 Development scorecard application in Indonesia^1^; the ACT Health citizen report card application in Uganda; and the World Vision application of citizen voice and action in Indonesia [32–34]. Summaries of interventions implemented, research methods, and key findings are provided in Appendix 1.

We then compared the checklist items reported in each of the worked example (see Appendix 2). Overall, none of the worked examples covered every item in the checklist in one paper. Looking across papers from a study and internal project documentation (based on discussion with study investigators), information for most, but not all elements, were reported or available. However, none of the worked examples provided keywords in the abstract (item 1c) or intervention components such as costs (item 5d), and all had no or limited discussion of harms (item 15) and of generalizability/external validity (items 16-17). All three worked examples contained information about the SA intervention description, as well as some details, if not all about the local context shaping the intervention. In our discussions, study investigators indicated that they did have additional information to report to fill some gaps, but either did not have space to include all information in one paper were still working on papers to fill in gaps.

### Checklist finalization

Based on the worked examples and our discussions, we removed repetitions within and between sections to streamline the checklist. We also divided out reporting on methods and results for quantitative and qualitative methods, to clarify what should be reported for each type of study. For the few items where none of the three examples had collected that information, we considered whether to retain the item. In all instances, we decided to retain the items because they had been identified as gaps in the umbrella review. For example, we retained items on content of the intervention because of its importance for interpretation of SA design, implementation, and evaluation.

### The final SAR4Research checklist (Brief version)

The final checklist contains six sections, each with several items that aim to ensure that reporting is robust, comprehensive and comparable across studies and contributes to the body of knowledge around SA. (Table 3). To make the checklist feasible to use, research teams with plans for multiple papers should consider what information to provide in each paper. For example, detailed information describing the evaluation and the intervention protocol can be cited in outcome papers. Thus, authors should consider, in advance, the sequencing of papers and grey literature reports, the depth of reporting on particular items in the checklist in each paper/report and provide cross citations among study papers and reports. Another option is to include clear and concise explanations for some checklist elements in an Annex (or more than one) in published papers, particularly as more journals allow for the inclusion of supplementary materials. These options will enable readers to develop a better understanding of the approach being evaluated, whether the evaluation design met the research objectives, and whether the results can be generalized to their own setting. Appendix 3 provides an explanation and elaboration of the final checklist.

**Table 3:** Final SAR4Research reporting checklist (expanded)

Section	Item description
**Title and Abstract**	**1a. Title**: Purpose (e.g., outcome evaluation) and study design (e.g., case study, realist evaluation) with commonly used terms. **1b. Abstract**: Structured summary of importance (e.g., health issue), study design with a commonly used term, methods (research and intervention), results (including participation rates), and conclusions. **1c. Keywords**: Keywords referring to social accountability and development outcomes
**Introduction**	**2a. Background**: Rationale for the study and how it contributes to what is known. **2b. Research objectives**: Specific objectives, research questions, or hypotheses. **2c. Theory of change**: Anticipated “mechanism” of action (I.e., theory of change, logic model, program theory or conceptual framework with intermediate and long-range outcomes). **2d. Setting or context**: Setting or context of the intervention, highlighting factors that influenced its design and implementation (e.g., geographic context, epidemiologic context, social context, political context).
**Methods**	**3. Study design** **3a. Design & development:** Who developed design & their role, description of study design with a commonly used term, explain how the design addresses objectives. As relevant, describe allocation (e.g., randomization/allocation) to conditions. **3b. Design changes:** Important changes after the study began (e.g., to design, participants, outcomes) and whether changes were part of an adaptive design.
	**4. Study participants and sample size** **4a. Eligibility criteria, sample size & selection.** - Inclusion and exclusion criteria for each group (intervention, comparison) of participants (e.g., individual, group, community, health system) for each data set (quantitative, qualitative, monitoring) included in analyses presented. Identify differences in eligibility for intervention and research participants, if relevant. - Planned sample size, for each data set presented. - Participant selection process for each data set presented. **4b. Data collection procedures:** Data collection, recording and storage procedures, for each data set presented (e.g., location, data collectors and whether they were blinded to assignment, study tools, types of questions, themes explored).
	**5. Social accountability intervention** **5a. Setting:** Where and when (month, year) implemented, and key characteristics that influenced design or expected outcomes. **5b. Social accountability intervention:** development, approaches & implementation: Background research (e.g., political economy analysis) and roles of developers (stakeholders & community). Name intervention tools, processes & components with terms used in literature. Specify scale (e.g., facility, subnational governance). **5c. Parameters:** Implementation stages, including who the intervention participants were and how marginalized groups were represented. **5d. Costs:** Estimate financial and other resources required for implementation
	**6. Overall goal and main objectives**: Define health and social accountability outcomes, including intermediate and longer-term outcomes along pathway of change.
	**7. Analytical methods** **7a. Quantitative analysis:** Statistical methods used to assess outcomes (e.g., compare groups on outcomes, test interactions, identify mediators), including methods to reduce biases, analyses to test pathways of change and any ancillary analysis (e.g., subgroup analyses, adjusted analyses etc.). **7b. Qualitative analysis:** Approach and analytic methods, including how saturation was determined, coding, reliability assessment, how themes were derived and analyzed. **7c. Implementation fidelity:** As relevant, describe methods used to describe and analyze fidelity to implementation plans. **7d. Triangulation**: As relevant, describe methods used for integrating/triangulating data
**Results**	**8. Implementation fidelity results**: As relevant, describe results.
	**9. Data collection results** **9a. Timing:** Provide dates (month, year) of recruitment, and all follow up periods, and if relevant why study was ended before planned. **9b. Quantitative data:** For each group (e.g., intervention, comparison) and each data set, provide numbers assigned, receiving the intervention, and analyzed for each outcome presented. Where possible, provide the number approached, screened and eligible prior to assignment, with reasons for non- enrolment. For each group, describe losses after assignment and reasons. **9c. Qualitative data:** For each group, numbers sampled and exposed to intervention.
	**10. Sample description**: Baseline sample characteristics, by group assignment (for each data set), highlighting important differences in analyses presented.
	**11. Main and other results** **11a. Quantitative results**: Results for each outcome for each group, providing estimated effect size and precision (e.g., 95% confidence interval). Provide results of other analyses, including mediational (test pathways) analyses, subgroup analyses and adjusted analyses **11b. Qualitative results:** Present major and minor themes for different groups/stakeholders. Describe diverse cases and provide supporting quotations. If relevant, describe how intervention influenced pathways of change or describe mechanisms of effects. Summary findings, interpretations, and theories generated. **11c. Triangulation results**: Results from combining datasets/mixed methods analyses.
	**12. Harms**: As relevant, all important harms or unintended effects in each group.
Discussion	**13. Limitations**: Address sources of bias, conflicts of interest, and changes in context (e.g., political instability) that occurred during implementation.
	**14. Generalizability**: Discuss generalizability (external validity, reliability, applicability), taking into account study population, intervention characteristics, length of follow-up, incentives, compliance rates, and specific site/contextual issues.
	**15.Interpretation**: Interpret all findings, balancing benefits and harms and considering other relevant evidence.
**Important information**	**16a. Trial registration/protocol**: As relevant, where study registered (provide link) and how protocol can be accessed. **16b: Declaration of Interest**: Sources of funding/support and other interests. **16c: Transparency**: Whether and where datasets are publicly available; whether/where ethical approvals were secured and key procedures.

## Discussion

We developed and tested a reporting checklist to ensure that design, implementation, and evaluation aspects of SA are more comprehensively and consistently reported by researchers in peer-reviewed articles. The motivation to develop the checklist stems from COP discussions around problems associated with reporting gaps, including our inability to identify patterns across studies about what works and what contextual factors are most important to consider in implementation. Although our review of reviews was not systematic, the reviews were consistent in gaps reported. The reviews included in our analysis and our own experience in SA suggest that the causes for these gaps are many, including cases where a robust evaluation was not planned, journal’s word limits, the volume of documentation and evaluation materials produced by study teams, and an underappreciation of process details in favor of major results. The SAR4 Research checklist may not address all these gaps, but aims to highlight the multiple factors that need to be better understood to build an evidence base for effectiveness of, and provide more guidance on, the design and implementation of SA interventions.

To the best of our knowledge, this checklist is the first attempt to address a gap in reporting for SA, and it is in line with other efforts to improve reporting, syntheses and use of findings from experimental studies, quasi-experimental studies and implementation research with the aim of improving and applying the evidence base around health programming [35–37]. For example, the WHO Programme Reporting Standards for Sexual, Reproductive, Maternal, Newborn, Child and Adolescent Health call for information on the context and stakeholders, recognizing the importance of both and the lack of attention to these elements in reporting guidelines for research studies [38]. In addition, assessments of implementation research to improve health programs identify the importance of adaptation and the need to understand when and how adaptations are made, thus suggesting the importance of documenting results of adaptive designs [37].

The final checklist aims to be flexible and versatile, irrespective of the SA interventions implemented and the evaluation design. We explored whether it would be feasible to report on all components through one article. However, in practice, each of our worked examples had several associated papers that documented the intervention design, implementation, and evaluation, with SAR4Research items spread across several papers and reports. Furthermore, research on SA is at its core interdisciplinary and, therefore, published across diverse peer-reviewed journals and grey literature reports. These journals’ word limits for research and review articles vary significantly, with health and biomedical field journal’s word limits being much tighter than in the social sciences. Given this insight, which is supported by our worked examples, the reporting checklist's purpose has shifted from being a checklist for a single paper to a checklist of information about a single study across a compendium of documents that summarize a single study. Where possible, we recommend that authors provide citations to other study papers when there is insufficient space to provide detail on each item in the checklist. This allows readers to understand the broader picture of the intervention and its effects.

Better reporting on SA is timely and relevant to support meaningful community engagement and strengthening accountability in health systems as part of the broader Universal Health Coverage movement and achievement of the Sustainable Development Goals [39]. Better reporting would help to enhance the interpretation of findings, as well as to compare results across settings – all of which are necessary to justify the long-term efforts needed to sustain and institutionalize accountability mechanisms.

### Limitations

Although we strove for comprehensive recommendations for reporting, we recognize several limitations in our methods. First, the checklist is intended for reporting in peer-reviewed articles, and thus may not meet the needs of implementers preparing monitoring or learning reports or for emergent SA interventions which often have less quantitative data to report. Furthermore, public health and clinical journals have a much shorter word limit than social science ones, representing an important barrier to reporting, particularly detail on intervention context and components. Thus, full reporting of the complexity of SA will require multiple papers/reports, often in different outlets. We did not assess the feasibility of using the checklist from the authors’ perspective, nor were we able to use it to determine what items to report on in different kinds of papers. Because a growing number of SA interventions are evaluated with mixed methods studies, modifying reporting recommendations for RCTs to meet the needs for reporting on other evaluations may lead to underreporting of important information about some study designs. Last, but not least, the worked examples used in our test of the checklist are not representative of the larger body of SA interventions. Smaller studies implemented locally, without sufficient resources could face different reporting challenges.

## Conclusions

Results of SA evaluations will be more useful to researchers and practitioners when study designs, context, and interventions are described fully and completely in manuscripts. The checklist aims to improve reporting, syntheses and use of findings from a range of study designs that can contribute to building the evidence base around SA, that can help inform future programming and more accountable health systems. This checklist will help authors identify and prioritize the relevant information to provide. Sufficient information will help researchers to identify the emerging findings and gaps in the literature that they might address with their own work. As with any reporting checklist, refinements are to be expected. The authors welcome feedback on the checklist as part of the wider effort to improve reporting and understanding of SA.

## Data Availability

Data sharing is not applicable to this article as no datasets were generated or analysed for the current study.

## Appendix 1

### Summary of Social Accountability Interventions and Outcomes, Worked Examples

This appendix provides an overview of each of the social accountability (SA) interventions, evaluations and results included in the worked examples. More details can be found in papers and reports from the studies [32–34].

#### Transparency for Development, Indonesia

Implemented by Harvard University, R4D, the University of Washington, J-PAL Southeast Asia, SurveyMETER, and a local civil society organization (PATTIRO), the project sought to empower rural communities to act to improve maternal and newborn health services. Using a community scorecard approach, community members received information on service-delivery problems and health outcomes and were mobilized to design and take actions to mitigate the problems. In follow-up meetings, community members tracked progress and identified new social actions. The evaluation used a randomized controlled design with cross-sectional surveys in 100 treatment and 100 control villages, as well as focus group discussions, interviews, systematic observations, and ethnographic studies. Although the evaluation showed no effect on primary and secondary outcomes, positive non-health outcomes such as increases in community participation and empowerment were noted.

#### ACT Health, Uganda

The Accountability Can Transform (ACT) Health, implemented by Civil Society Organizations and Goal Uganda, provided information about the quality of services to community members and providers, who in turn formulated action plans to improve service delivery. The program had three components: 1) provision of citizen report cards to health providers; 2) facilitation of separate meetings with providers and citizens to develop action plans; and 3) interface meetings to discuss next steps. The evaluation used a randomized design with 376 health facilities randomly assigned to one of four groups: all three components; components 1 and 2; component 3; and no intervention. Although survey data showed no improvements in utilization rates or health outcomes in any of the intervention arms, the communities that received the full program had “marginally better-quality care” and higher satisfaction with services compared to the non-intervention community..

#### World Vision, Indonesia

The ‘Citizen Voice and Action for Government Accountability and Improved Services: Maternal, Newborn, Infant and Child Health Services’ project was implemented by Wahana Visi Indonesia in three districts and took place in two stages with 30 villages in each stage. The project used World Vision’s ‘Citizen Voice and Action’ (CVA) approach in which village-level facilitators were trained to mobilize other villagers to assess health services against official and villager-determined standards. Plans for service improvement along with advocacy efforts with high-level officials were initiated. Using a realist methodology, the evaluation drew from 1) household surveys; 2) surveys with officials and providers; 3) assessments against government standards; 4) community scorecards; 5) most significant change stories; and 6) program administrative data. Household surveys revealed statistically significant increases in respondents’ knowledge of services provided and health service standards. The results from the surveys with officials and providers showed improvements in the ability to name the services required by standards and reports of improvements in the quality of services at multiple levels (health posts, birthing centers and health centers). There were also positive changes in the scorecard data, evidencing greater community satisfaction with services provided.

## Appendix 2

### Annex Draft Checklist Items Reported in Each Case Study

**Table:**

***Item***	**T4D**		**ACTHealth**		**World Vision**	
	Papers & reports	Project files	Papers & reports	Project files	Papers & reports	Project files
**Title & Abstract**						
1a Title: Purpose & study design	*Yes*		*Yes*		*No*	
1b Abstract: Importance, study design, methods, results, and conclusions	*Yes*		*Incomplete*		*No*	
1c Keywords: Social accountability & health	*No*		*No*		*No*	
**Introduction**						
2a Background and rationale	*Yes*		*Yes*		*No*	
2b Research objective/questions	*Yes*		*Yes*		*Yes*	
2c Theory of change	*Yes*		*Yes*		*Yes*	
2d Role of context	*Yes*		*Yes*		*No*	*Yes*
**Methods**						
3. Study Design						
3a Study design & how addresses objectives	*Yes*		*Yes*		*Yes*	
3b Who designed study & how	*Yes*		*No*		*Yes*	
3c Changes after study began	*No*	*Yes*	*No*		*No*	*Yes*
3d Trial registration & protocol	*No*	*Yes*	*Yes*		*No*	
4. Study participants and sample						
4a Inclusion/ exclusion criteria & recruitment	*Yes*		*Incomplete*		*No*	*Yes*
4b Unit of assignment	*Incomplete*		*Yes*		*NA*	
4c Participant selection & representativeness	*Yes*		*No*		*No*	
4d Data collection settings	*Yes*		*Yes*		*Yes*	
4e Data collection methods	*Yes*		*Yes*		*Incomplete*	
5. Intervention description						
5a Intervention strategies named	*Yes*		*Yes*		*Yes*	
5b Description of implementation stages, representation of marginalized groups	*Yes*		*Yes*		*No*	
5c Adherence to delivery protocols	*No*	*Yes*	*No*		*No*	*Yes*
5d Financial and other resources required for implementation	*No*		*No*		*No*	
6. Overall goal & objectives						
6a Accountability outcomes	*Yes*		*Yes*		*Yes*	
6b Outcomes for sub-groups	*Yes*		*Yes*		*Incomplete*	
6c Changes to outcomes after study begins	*Yes*		*No*		*NA*	
7. Analytical Methods						
7a Statistical methods	*Yes*		*Yes*		*No*	
7b Qualitative methods	*No*		*NA*		*Yes*	
7c Methods used to describe intervention implementation	*No*		*Incomplete*	*Yes*	*No*	*Yes*
7d Methods for integrating and triangulating data	*Yes*		*NA*		*Yes*	
7e Methods for examining interactions and mediators	*No*		*Yes*		*No*	
Results						
8. Implementation Analysis	*Yes*		*Incomplete*	*Yes*	*No*	*Yes*
9. Data collection outcomes						
9a Numbers assigned, receiving intervention, and analyzed	*No*		*Yes*		*No*	
9b Numbers sampled and exposed to the intervention	*Yes*		*NA*		*Yes*	
10. Recruitment						
10a Dates of recruitment and follow-up	*Yes*		*Yes*		*Yes*	
10b Why the study was stopped	*NA*		*NA*		*NA*	
11. Baseline data	*Yes*		*Yes*		*No*	
12. Numbers analyzed						
12a Number in each analysis and whether intent to treat	*Yes*		*Yes*		*Yes*	
12b Description of how noncompliers were treated	*NA*		*Yes*		*NA*	
13. Outcomes and estimations						
13a Estimated effect size and precision	*Incomplete*		*Yes*		*Yes*	
13b Major and minor themes	*No*		*NA*		*Yes*	
14. Ancillary Analyses						
14a Results of subgroup and adjusted analyses	*Yes*		*Yes*		*No*	
14b Results from mixed methods analysis	*Yes*		*NA*		*Yes*	
15. Harms & unintended effects	*No*		*No*		*No*	*Yes*
Discussion						
16. Limitations	*No*		*No*		*No*	
17. Generalizability						
17a Generalizability for RCTs	*No*		*No*		*NA*	
17b Generalizability for quasi-experimental	*NA*		*NA*		*No*	
17c Generalizability for qualitative studies	*No*		*NA*		*No*	
18.Interpretation	*Yes*		*Yes*		*Yes*	
Important Information						
19. Registration	*No*		*Yes*		*No*	
20. Protocol	*Yes*		*No*	*Yes*	*No*	
21. Declaration of Interest	*Yes*		*No*		*No*	
22. Stakeholder Involvement						
22a Involvement of intervention developer in the study	*No*		*No*		*Yes*	
22b Stakeholder involvement in study design	*Yes*		*Yes*		*Yes*	
22c Incentives	*Yes*		*Yes*		*No*	*Yes*
23. Transparency						
23a Datasets publicly available	*No*		*No*		*No*	
23b Statement of ethical approvals	*No*		*Yes*		*No*	

## Appendix 3

### Explanation and elaboration of the final SAR4Research Checklist

This Appendix provides additional details about how the checklist items should be applied for reporting evaluations of social accountability (SA) interventions. If the in CONSORT-SPI was sufficient for SA, we summarized the requirement and did not add detail [5, 10].

### Title and Abstract

#### 1a. Title

Identifying the purpose of the study and the study design in the title will increase the likelihood that the article will be indexed correctly. Authors should include the intervention’s name and the health or development focus (e.g., maternal health) to facilitate the identification of relevant papers. Many SA evaluations do not use randomized or quasi-experimental designs and some aim to understand implementation processes. Thus, it is important to add the purpose (e.g., outcome assessment, process evaluation) in the title.

#### 1b. Abstract

Because abstracts may be used to determine if a paper meets a reader’s needs, a structured abstract should refer to the importance of the study, study design, methods, results, and conclusions. Many SA studies are not trials, and so the abstract should include the study design with commonly used terms (e.g., case study, realist evaluation) and should identify participants (type, numbers) and outcomes at all levels (e.g., community, organization, individuals). Because of the complexity of accountability interventions, it would be useful to specify key points in the causal pathways in the program’s theory of change.

#### 1c. Keywords

Keywords are used for indexing and make it easier for others to find papers. Keywords should include keywords for SA and for the health area.

### Introduction

#### 2a. Background

The introduction should describe the rationale for the study, and how it contributes to what is reported in the literature. Guided by what is known and assessments to develop the intervention (e.g., political economy analysis), the introduction should describe the health, governance and accountability issues addressed, as well as how the study will contribute to the understanding and implementation of SA interventions.

#### 2b. Research objectives

Research objectives, including research questions or hypotheses and expected effects at each level (e.g., community, system, organization, individual) should be described, including whether and how the study will assess pathways of change*.* When evaluation studies are not able to identify all outcomes or effects sizes in advance, research questions should detail what outcomes could occur, and the direction of effects. Process evaluation reports should identify processes and implementation parameters addressed.

#### 2c. Theory of change

Although CONSORT-SPI includes the mechanism of action in “research objectives”, we made this (and 2d) separate items. SA interventions seek to change power and decision-making dynamics and for participants to generate solutions that improve the health system functioning and health outcomes. They are context-driven and more complex than many other social or psychological interventions. The focus on community engagement and empowerment suggests that details of activities and processes may not be known in advance, which in turn point to the importance of identifying, in advance, expected outcomes along a change pathway that links intervention activities to intermediate and longer-term modifiable individual, organizational and community/social processes and outcomes. Tables or graphical depictions may help convey the casual pathways embedded in the theory of change. These explanations may benefit from attention to theory used to develop interventions [17, 22, 23].

#### 2d. Setting or context

Understanding the context is central to SA; it influences implementation outcomes and interpretation of results. The CICI recommendations provide questions to consider when documenting the context (i.e., what are the theoretical underpinnings? how does this theory interact with the context? and how does this theory interact with the intervention?) [31]. Aspects of the context, at national, sub-national or local levels, to consider include: *geographical* (geographical barriers to accessing services); *epidemiological* (distribution of health outcomes and determinants); *socio-cultural* (behavioral patterns, values, ideas, social roles, etc.); *socio-economic* (access to resources); *ethical* (morality, norms); *political* (distribution of power, assets, and rules governing interaction) [31]. In addition, we suggest considering the micro context (e.g., individual, household, and facility characteristics), which is often the locus of deconstructing power relationships. Such information, in addition to documentation of or linkages to existing SA activities in the site, will help readers determine whether an intervention is relevant for their context and adaptations needed.

### Methods

Specifying the study’s methods allows readers to assess the suitability of the study design to answer the research questions and interpret the results. Most recommendations, including CONSORT-SPI, assume that a randomized controlled trial or quasi-experimental design is used to evaluate behavioral, psychological, and social interventions. Although reporting guidelines allow for complexity in methods (e.g., for more than one unit of assignment and analysis), they rarely assume that multiple datasets and methods or that an adaptive design is used. Thus, SA studies may require more detailed descriptions of methods in one or across a set of papers.

#### 3a. Study design & development

Manuscripts should describe and explain the choice of the study design, including the purpose (e.g., benefits relative to standard of care). If relevant, the details of assignments (or randomization) of individuals or clusters (e.g., organizations, health facilities, communities) should be reported, and an explicit statement should be provided if the unit of analysis differs from the unit of assignment. Because many SA studies do not use randomized or quasi-experimental designs, it is important to identify the design with commonly used terms (e.g., realist evaluation, case study, process tracing, outcome harvesting/mapping, qualitative comparative analysis), and explain how the design addresses the objectives. For example, does the study assess how intermediate outcomes contribute to health outcomes or to understand implementation processes? Those who participated in developing the study design should be identified and their roles described (particularly for participatory research), so readers understand the different perspectives that contributed to the design and interpretation of the data. In addition, the methods should describe from whose perspective data were collected and analyzed [6, 15, 16].

#### 3b. Design changes

Because evaluations of SA interventions sometimes use adaptive designs, the manuscript should state whether such a design was used and how methods were expected to identify the need for adaptations. Whether an adaptive design was used or not, all changes and their potential effects on outcomes should be described.

#### 4a. Eligibility criteria, sample size & selection

Manuscripts should identify eligibility criteria (i.e., inclusion/exclusion criteria) for all participants (e.g., individuals, groups/organizations, facilities, health systems) for each dataset (i.e., quantitative, qualitative, and monitoring) used in analyses presented; this will help readers assess the generalizability of results. Inclusion and exclusion criteria for all types of participants should be provided, even if all units do not contribute data (e.g., communities where interventions carried out). If criteria for participation in the intervention and data collection differ, those differences should be explained. Planned sample sizes should be stated, along with justifications (e.g., statistical power, complexity of design, cost limitations, etc.). When used, purposive or theoretical sampling methods should be described

#### 4b. Data collection procedures

Describe all data collection procedures for all groups (i.e., intervention and comparison; individuals, groups communities) for each dataset used. This should include such things as who recruited participants and conducted interviews, from where participants were recruited and data were collected, whether interviewers were blinded to allocation , experience and training of data collectors, tools used for data collection (e.g., interview guides, ACASI), and storage and confidentiality of data.

#### 5a. Social accountability implementation setting

Descriptions of the settings, locations, and timing of the intervention, and the comparison condition if relevant, allows readers to determine the relevance to their situation. When reporting on SA, describing the *s*etting and locations should be more specific than the context in 2d, and include items such as actual location (e.g., clinic, community venue) where activities occurred and the timing of activities. The theory of change and the information presented on the context (in section 2) can guide what is reported here. When a comparison group is included, the description of the setting and location should draw attention to differences that might influence outcomes.

#### 5b. Social accountability intervention: development, approaches and implementation

Participants, the organizations they represented, the reason they were included, and the role they played in developing the intervention should be included. Any effort to include stakeholders and community members in development should be described. Sufficient detail on the intervention(s) being tested, as well as the comparison if relevant, are required to understand, synthesize, and replicate interventions. Details about those delivering interventions, such as qualifications and training should be included. Such information provides insight into the relationships, time and materials needed to develop or adapt the intervention.

SA initiatives use an array of approaches (e.g., community mobilization, community scorecards, expenditure tracking). It is important to identify all items, the rationale for inclusion, and how they are expected to influence outcomes. In addition, descriptions should also state the details about: start-up actions (e.g. stakeholder engagement, formative work, training and sensitization ); the level and timing of the intervention (funded short term, system-oriented, grassroots); intended recourse process (mechanisms for recourse); internal and external actors (individuals & organizations) involved in decision-making , implementation (e.g., providers, advocates, meeting facilitators (highlighting whether INGO or community members), intended post-implementation activities (inclusive of an exit strategy and community handover); and linkages with other accountability efforts (e.g. government mechanisms or social movements). Documentation of whether and to what extent the intervention evolved over time should be included. Authors should identify additional resources where more information on the intervention can be found (e.g., non-published reports, project websites). This includes implementation protocols, training manuals, tools, and materials.

#### 5c. Implementation parameters

The stages of the implementation process passed through should be described. For instance, how does the implementation process interact with the setting and the context? And how does it interact with the intervention? A key component of this is whether and how marginalized groups were included in the intervention, what role did they play, and how were their perspectives included and elevated? In addition, if a pilot was carried out, explain how the pilot results influenced implementation in the study context [28].

#### 5d. Costs

Given the complexity of SA and the increased attention to cost-effectiveness, estimates of the resources required for implementation should be described. Costs should address “who does” (i.e., costs related to participation) and “who pays” (i.e., actual expenses for this such as staff, tools and materials development and use, etc.) [27, 28]. Documenting number of activities, facilitators time and costs, each of the activities, and technical assistance will provide for a fuller sense of costs.

#### 6. Overall goal and main objectives

Outcome definitions, derived from research questions, hypotheses or objectives, should include the construct or domain, the measure, and the metric used. SA evaluations often have multiple outcomes; all should be described, including health (e.g., service use, voluntary contraceptive use), health systems (e.g., improved quality, health system responsiveness), SA and community (e.g., empowerment, collective action, social norms) and governance (e.g., collaboration) outcomes. A discussion of the relationships among the outcomes should be described, for example, what is the pathway of change. In addition, any and unintended outcomes should be discussed.

#### 7a. Quantitative analysis

A full description of the analytic methods used for the quantitative data allows for study replication, as well as a better understanding of the results. For each outcome (or set of outcomes), this should include variables used (e.g., individual items, scales created) and types of analyses (e.g., logistic regression), including adjustments and methods to reduce biases. If the unit analysis differs from the unit of assignment, the statistical methods used to account for such differences should be reported. If there is only one group (i.e., intervention), a description of the analytic methods should include similar information, with a focus on how potential sources of biases were addressed. Additional factors to consider include missing data (e.g., extent of missing data, how it was handled), sub-group analyses (e.g., how subgroups were constructed, whether analysis is exploratory or confirmatory), mediational analyses to understand processes of change (e.g., identifying intervening variables and measured, specify analytic procedures) and whether/to what extent data from different types of participants are triangulated to assess outcomes.

#### 7b. Qualitative analysis

Complete reporting of qualitative analytic methods allows for a better understanding of the nature and scope of the analysis. This should include descriptions of assessment of saturation, translation and transcription (including verification), timing of coding/analysis (e.g., as data collected or when all data collected), whether coding was automated or manual, approach to coding (e.g., deductive, inductive), code book development and refinement, a description of the coding tree, number of coders and procedures for assessing inter-coder agreement, and software used. Given the participatory nature of SA, it is important to report whether study participants provided feedback in the process. The COREQ recommendations for reporting qualitative studies provide more detail.(8)

#### 7c. Implementation fidelity

Within CONSORT-SPI, there is little attention to process or project monitoring data. However, for many SA interventions project monitoring data are used in evaluations or may be used as the basis for one paper/report. Thus, process data should be described, including who collected data, what was measured, and the purpose of using the measures in the analyses presented (e.g., to describe fidelity to implementation plans, to better understand / contextualize outcomes).

#### 7d. Triangulation

Because many evaluations only use data from intervention beneficiaries, most reporting guidelines do not address whether and how to report on analyses to triangulate data. However, many SA interventions use data from more than one group of respondents or one data source, and so it is important to explain how data are used together to assess the effects of or better understand the intervention. The purpose of each type of data should be explained (e.g., outcomes for whom) and how it relates to understanding the causal pathways (e.g., changes in service delivery contribute to changes in health outcomes).

### Results

#### 8. Implementation fidelity results

The results of the implementation or process analysis should be summarized, including the extent of fidelity to implementation plans, as well as facilitators and barriers to implementation (e.g., external conditions occurring that might have influenced the intervention. In addition, consider reporting on how the implementation process interacts with the contextual features and how the intervention evolves as a result (e.g. plans change through repeated consultation with local stakeholders).

#### 9a. Timing of data collection results

Provide dates for all data collection (in months and years), as well as dates of implementation of the intervention. This helps readers set the intervention and results in context (e.g., any world-wide, county or local events, such as a pandemic, national elections, etc.), as well as information on the length of implementation needed for effects and the potential duration of effects. If the intervention was stopped prior to the planned end date, provide information (month/year) when stopped and a rationale or explanation.

#### 9b. Quantitative data collection results

For the intervention, and comparison groups if relevant, provide the number of participants for data collection and for implementation for each dataset presented in analyses. Because attrition at different stages bears on conclusions, manuscripts should provide the number approached, the number screened, the number eligible, the number who enrolled and were assigned, and the number who completed baseline data collection. In addition, loss to follow up and other exclusions after assignment should be reported for each group (intervention/comparison) and each type of data, and should include reasons for loss to follow up. If non-compliance or contamination are issues, provide numbers of cases and reasons.

#### 9c. Qualitative data collection results

For qualitative data, the manuscript should provide information on the number approached, the number screened, the number eligible and the number who participated, for both intervention and comparison groups [8]. In addition, if measures were taken to assess saturation, the numbers at which saturation were reached should be provided.

#### 10. Sample description

A table providing characteristics of participants (at all levels) for intervention, and comparison groups if relevant, at baseline should be provided. This should include baseline data on outcomes, key intermediate variables, and characteristics that might contribute to outcomes (e.g., socio-economic status). Data should be included for participants in qualitative data collection, to the extent that characteristics may influence their responses to the interviews (e.g., community leaders vs community members; men vs women).

#### 11a. Main and other quantitative results

For each outcome, point estimates for each group (intervention and comparison), the magnitude of the difference, and the precision of the estimate (e.g., confidence intervals) should be provided. The effect size can be presented in different ways (e.g., odds ratio, risk ratio, mean difference) depending on the analyses and how outcomes are measured (e.g., categorical, continuous). Analyses should be presented for all types of participants (communities, organizations, individuals), and should include results from the “most adjusted models”, results from analyses to identify pathways of change, and sub-group or ancillary analyses performed.

#### 11b. Main and other qualitative results

Reporting on qualitative data should include a description of major and minor themes regarding the intervention and its results, as well as any analyses that point to how the intervention might work (i.e., effects had, timing or effects relative to each other, etc.). Differences by sub-group, divergent cases and supporting quotations should be provided.

#### 11c. Triangulation results

To the extent that, multiple sources of data were included and analyzed together, those analyses should be presented. For example, if qualitative data support or explain quantitative findings, such explanations should be included in the results.

#### 12. Harms

Interventions can produce beneficial and harmful unintended effects. Any harms should be reported, including the nature of the harm, whether it was anticipated, and how it was assessed (e.g., quantitative data, qualitative data) and addressed. Because SA approaches rely on participatory processes and address power dynamics (e.g., decision-making around health services) in the community, it is important to report social harms (e.g., stigmatization) and benefits (e.g., women’s empowerment).

### Discussion

#### 13. Limitations

The manuscript should describe the strengths and limitations of the study design, considering such things as potential biases, precision of quantitative estimates of effects and fidelity to the intervention and to SA principles. Other limitations may include conflicts of interest and changes in the implementation context (e.g., disruptions due to pandemic, unanticipated changes in operational or other policies).

#### 14. Generalizability

The discussion should address whether and to what situations the results are generalizable. Issues to consider include whether the intervention and comparison groups were randomized or assigned, imbalances between study groups (e.g., in SES of participants), recruitment processes, eligibility criteria, outcomes assessed, and implementation parameters (e.g., fidelity to the intervention as planned). Given the nature of SA interventions, generalizability should consider the context and setting of the intervention, as well as dynamics within and among the community, organizations and individuals.

#### 15. Interpretation

Interpretation of the results should be framed in light of the objectives, research questions or hypotheses, and may include explanations for results not in line with expectations or any harms (anticipated or unanticipated), how results contribute to the literature (e.g., other findings, theory of change guiding intervention) and practical implications (e.g., dissemination, resource needs, further testing). Because of the context sensitive nature of SA interventions, it is particularly important to interpret the results in light of the theory of change (or mechanisms of action) by which the intervention was expected to work, and the role of context and setting in shaping the results and key implementation barriers and facilitators.^21^

### Important Information

#### 16a. Trial registration/protocol

As relevant, provide information (link, reference) to the trial registry and where the protocol (particularly if the study was not registered) can be accessed.

#### 16b. Declaration of Interest

Provide information on sources of funding, and the role of funding sources in development or implementation. Identify other conflicts of interest

#### 16c. Transparency

State whether data are publicly available, and if so where and how they can be accessed. Provide information on human subjects protections, including when/where ethnical approval was obtained and key processes and procedures to protect participants rights and privacy with respect to the research process.

## Abbreviations

ACASI: Audio computer-assisted self-interviewing
ACT: Accountability Can Transform (ACT)
COP: Community of Practice on Measuring Social Accountability and Health Outcomes
CVA: Citizen Voice and Action
INGO: International Non-Governmental Organization
J-PAL: Abdul Latif Jameel Poverty Action Lab
R4D: Results for Development
SA: social accountability
SAR4Research: Social Accountability Reporting for Research
SES: Socio Economic Status
T4D: Transparency for Development
WHO: World Health Organization

## Footnotes

1 The program was implemented in five countries (Indonesia, Tanzania, Ghana, Malawi and Sierra Leone) but the checklist tested reports from Indonesia only.
